# Hospital Door Handle Design and Their Contamination with Bacteria: A Real Life Observational Study. Are We Pulling against Closed Doors?

**DOI:** 10.1371/journal.pone.0040171

**Published:** 2012-10-15

**Authors:** Hedieh Wojgani, Catherine Kehsa, Elaine Cloutman-Green, Colin Gray, Vanya Gant, Nigel Klein

**Affiliations:** 1 School of Construction Management & Engineering, Reading University, Reading, United Kingdom; 2 Infectious Diseases and Microbiology Unit, Institute of Child Health, UCL, London, United Kingdom; 3 Department of Microbiology, University College London Hospitals NHS Foundation Trust, London, United Kingdom; New York State Health Department and University at Albany, United States of America

## Abstract

**Objective:**

To determine whether microbial contamination of door handles in two busy intensive care units and one high dependency unit was related to their design, location, and usage.

**Design:**

Observational study of the number of viable bacteria on existing door handles of different design at defined entry/exit points with simultaneous data collection of who used these doors and how often.

**Setting:**

Two busy specialised intensive care units and one high dependency unit in a tertiary referral NHS neurological hospital.

**Main outcome measures:**

Surface bacterial density on door handles with reference to design, location, and intensity of use.

**Results:**

We found a significant correlation between the frequency of movements through a door and the degree to which it was contaminated (p = <0.01). We further found that the door's location, design and mode of use all influenced contamination. When compared to push plate designs, pull handles revealed on average a five fold higher level of contamination; lever handles, however, displayed the highest levels of bacterial contamination when adjusted for frequency of use. We also observed differences in contamination levels at doors between clinical areas, particularly between the operating theatres and one of the ICUs.

**Conclusions:**

Door handles in busy, “real life” high acuity clinical environments were variably contaminated with bacteria, and the number of bacteria found related to design, location, mode and frequency of operation. Largely ignored issues of handle and environmental design can support or undermine strategies designed to limit avoidable pathogen transmission, especially in locations designed to define “thresholds” and impose physical barriers to pathogen transmission between clinical areas. Developing a multidisciplinary approach beyond traditional boundaries for purposes of infection control may release hitherto unappreciated options and beneficial outcomes for the control of at least some hospital acquired infections.

## Introduction

Healthcare Acquired Infections (HCAIs) continue to threaten the quality of patient care. The human and financial cost to individuals, healthcare organisations and society is considerable, approximating to £1.5bn per annum in the UK alone [1]. Governments and healthcare providers have intervened with a variety of measures, guidelines and regulations designed to control HCAIs [2]. Accordingly, much progress has been achieved with interventions relating to hand hygiene, strict infection control monitoring and cleaning regimes. Further progress is likely to follow from the identification of other potentially important contributors to HCAI, such as the design of the hospital itself and how this determines people's movement and behaviour within it [3]. There is increasing interest in the design of healthcare establishments, driven by issues of efficiency in both primary and secondary care facilities [4]. Hospital design is even more relevant for maintaining care quality in the face of space constraints, higher patient acuity, shorter lengths of inpatient stay and financial pressures. The operational challenges set by these agendas are substantial, and consideration should also be given to how these design variables might present, or prevent, opportunities for transmission of pathogenic organisms. Little data exist to inform how hospital design might impact on the potential for HCAI transmission [5]. With this in mind, built-environment experts, clinicians, microbiologists, and statisticians came together to examine possible relationships between defined elements of hospital design, behaviour and environmental contamination.

Specifically, we sought to generate data relating to microbial contamination on door handles and how this might be related to factors relating to their design and use. We selected three high acuity environments for study as these are known to act as hotspots for HCAI transmission [6]. Finally, we suggest using relevant findings as evidence to generate novel strategies for infection control.

## Methods

This was an observational study of a nine-bedded surgical intensive therapy Unit (SITU), a newly refurbished four-bedded medical intensive therapy unit (MITU) with a side room, and a four-bedded high dependency unit (HDU), all located in close proximity to each other on one floor of a busy urban hospital. We obtained waivers from our ethics Committees for the work as the study neither involved patient contact, nor was disruptive to patient care. Studies were carried out in a six month period between 2008 and 2009. We gathered information relating to ward layout, which way the doors into, out from, and within the units opened, how often they were used, by whom, the door handle design, and finally contamination density by potentially harmful microorganisms.

**Figure 1:**
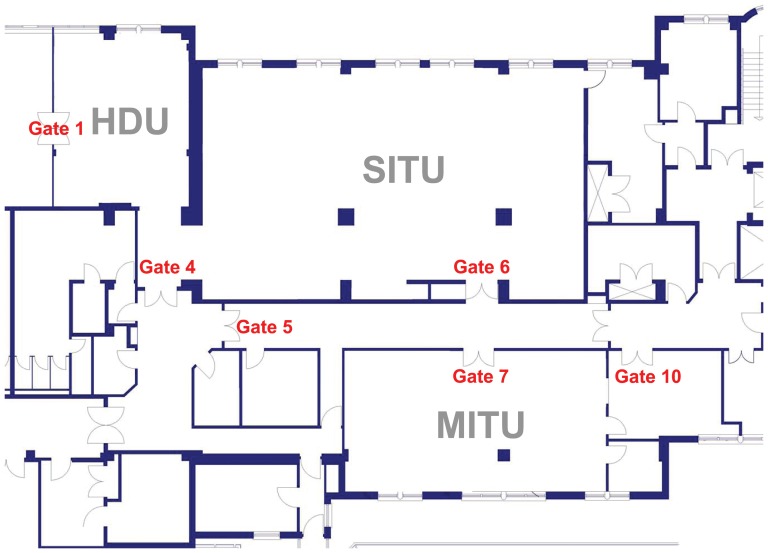
Plan of the units. Gates were defined as those thresholds across which individuals travel. Gate numbers were not consecutive, as some gates had no doors. Gates and doors (when present) were numbered using the same numbering system.

Figure 1 shows a plan of the units. Gates were defined as those thresholds across which individuals travel. Gate numbers were not consecutive, as some gates had no doors. Gates and doors (when present) were numbered using the same numbering system. Gate 1 identified the door connecting the HDU to the operating theatres zone; Gate 4 the main entrance to the SITU and HDU; Gate 5 the doorway to the main corridor separating SITU from MITU; Gate 6 the second entrance into the SITU; Gate 7 the main entrance to MITU, and Gate 10 one of the entrances to the only side room of MITU which opens directly into the main corridor. This side room could also be accessed through MITU.

**Figure 2:**
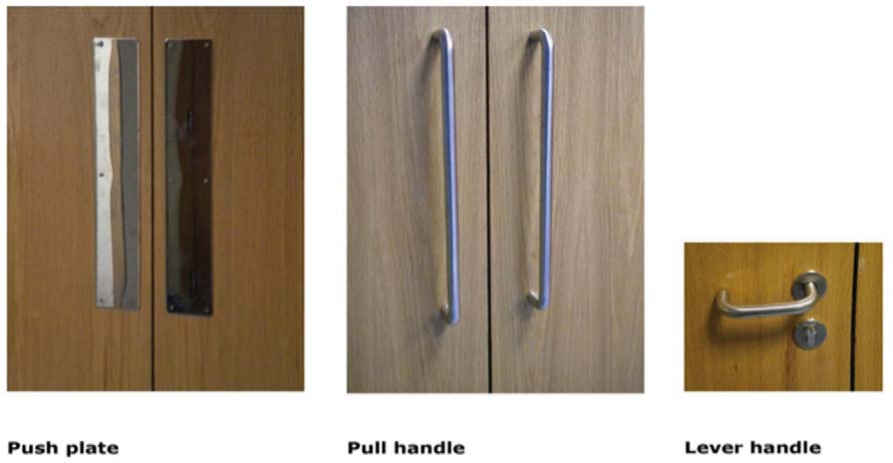
Images of the door handle types.

Doors with push plates always had a fixed pull handle on the other side. The direction of push or pull varied from door to door. Gates 4 and 6 were furnished with a pull handle to enter the unit, whereas Gates 5 and 7 used a pull handle to leave the unit. We observed staff and visitors for at least three days for all six gates. The doors at gates 1 and 10 had lever handles while the other four (Gates 4, 5, 6 and 7) were double leaf doors designed to be pushed on one side and pulled on the other. Accordingly, the doors we studied had three different designs: flat rectangular metal plates on the push side of the double doors, longitudinal fixed door handle bars on the pull side of the double doors and a short horizontal lever handle on both sides of gates 1 and 10. These different designs are shown in figure 2.

### Observing people's movement

We watched where people moved to and from and recorded our observations. We were careful to allow a “run-in” period of sham observation of three weeks in order to minimise any bias which the observation process itself might trigger. A single movement was defined as one individual crossing the threshold of any gate as defined above and the locations of which are illustrated in Figure 1. We monitored all movements through all gates in the three units on a daily basis from 10:30 to 13:00 and from 14:30 to 17:00. Individuals were assigned to one of several groups, namely staff local to the ward, other hospital staff, patients, and their visitors.

### Microbiology

Microbiological surveillance data were collected at the same time as handle usage using Tryptone Soy Agar (TSA) Rodac impression plates with a surface area of 16.7 cm^2^. We chose Rodac plates rather than a swabbing technique as it reduces variation relating to swab material type and swabbing technique. The plates were read after 48 hours' incubation for Total Viable Counts (TVCs). We sampled both door handles and door plates. These were cleaned thoroughly with 70% isopropyl alcohol wipes immediately before the start of the movement observations and swabs taken to ensure the handles and plates were free from bacteria. We repeated the sampling at the same sites following a 150 minute observation period. This was found to be sufficient for observing substantial door usage whilst practical for continuous observation by a single worker. We developed consistent sampling techniques whereby we sampled a 100 cm^2^ area at the centre of the door push plates, or a rotation of the Rodac impression plates around the vertical centre of the fixed vertical door handles. This was repeated twice a day to straddle both morning ward rounds and afternoon visits by relatives, and for three days.

### Statistical analysis

Data were analysed using SPSS 16.0 for Windows. Initial data analysis demonstrated the data distribution to be non-Gaussian. Accordingly, we used Spearman's Rho Product Moment test to determine the relationship, if any, between movements through various doors and microbial densities. We used one way ANOVA for least significant difference analysis to establish the significance of any difference between means. After correction for extreme values, we used the Pearson Product Moment test parametric analysis. We expressed results as means ± standard deviation. Values were considered significant for p values of less than 0.05.

## Results

### Bed Occupancy and Movements

We observed ward traffic for periods of seven consecutive days, during which there were no to four patients present in the four bedded HDU; five to seven patients in the nine bedded SITU; and three to four patients in the four bedded MITU. We recorded up to 241 movements across a gate in 150 minutes at a time when only six out of nine beds were occupied. Staff based on that ward were responsible for 50% of all movements through this particular gate. Accordingly, various staff members had to exit and/or enter the unit about 120 times over a two and a half hour period. Table 1 displays the total number of movements according to category of building user over a seven day observation period. These data demonstrate large variations of traffic across doorways, which were related to location and time, but not direction. Ward and hospital staff generated the majority of these events. Movements through the main entrances to the ITUs (Gates number 4 and 5), constituted almost 47% of all movements.

**Table 1:** Various Types of Users Passing Through Each Gate.

Door No	Ward Staff		Other Staff		Visitor		Patient		Total
	No.	%	No.	%	No.	%	No.	%	
1	41	66%	21	34%	0	0%	0	0%	62
4	381	50%	262	31%	146	18%	8	1%	797
5	249	36%	332	47%	109	16%	6	1%	696
6	296	51%	219	38%	58	10%	3	1%	576
7	580	57%	402	39%	37	4%	5	0%	1024
10	36	73%	13	27%	0	0%	0	0%	49

No. denotes the number of individuals moving through a gate. This is then expressed as a percentage broken down by their reason for being on the ward.

### Door Handle Design, Movement density and Microbial Growth

Microbial growth from Gate 6 was on many occasions either confluent, or too numerous to count, as was one sample from Gate 5. There was little effect of sample timing on TVCs apart from Gate 6, where the afternoon samples were consistently found to be greater than 300 or were confluent.

**Figure 3:**
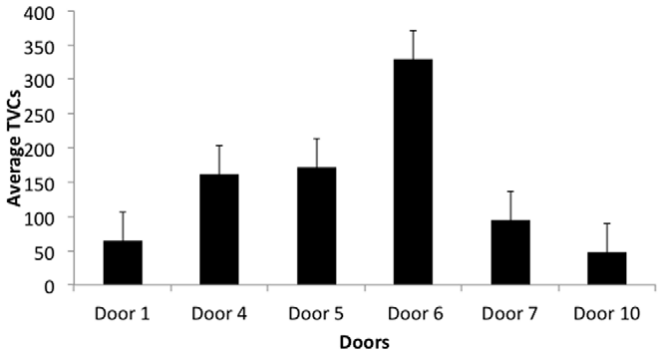
Average Total Viable Counts +/−1 Standard deviation retrieved from both sides of each door.

Figure 3 shows the considerable range of average TVCs retrieved from both sides of each door. We occasionally detected confluent or near confluent bacterial growth on door handles in the context of low levels of traffic (Gates 1 and 10). These exceptions can only be explained by less frequent contact with highly contaminated hands. When these heavily contaminated samples were excluded, a significant correlation between movement density and TVCs emerged (<0.01). Low traffic density was associated with low TVCs for Gates 1 and 10 and the more heavily used doors at Gates 4, 5, 6 and 7 were more contaminated. Further analysis of the pattern of contamination in the more heavily used doors indicated that other factors were contributing to microbial contamination.

Traffic density heading either in or out of the doors was balanced and was not influenced by the door handle design. Analysis of individual and average TVCs for each type of door handle, however, revealed that bacterial load on pull handles was consistently higher than that on the push plates located on the other side of the door. This narrowly failed to reach statistical significance (p = 0.053). Further analysis relating to handle type revealed that lever handles had the highest ratio (6.38 TVCs/movement), followed by Pull handles (2.24 TVCs/movement), which were in turn nearly double that of the Push plates (1.20 TVCs/movement). Interestingly, the ratio of TVCs/movements on the lever handles located on the inside of the doors used to exit from the side room and HDU was much higher than the corresponding handle on the other side of the door (Table 2). The table also shows that pull handles had a higher ratio of TVC per movement than the push handles.

**Table 2:** Ratio of TVC/Movement for Each Type of Handle.

Gate No.	Going In	TVC/Movement in	Going out	TVC/Movement out	TVC/Movement
1	Handle	0.43	Handle	8.56	4.63
4	Pull	1.82	Push	0.49	1.18
5	Pull	2.63	Push	1.29	1.97
6	Pull	5.44	Push	0.99	3.27
7	Push	0.62	Pull	0.76	0.69
10	Handle	1.57	Handle	14.52	8.56

## Discussion

We found a relationship between how often and how many people cross door thresholds and the number of bacteria deposited on door handles. This finding supports the requirement for hand hygiene whenever hospital thresholds are crossed [7]. These critical moments in potential microbial transmission are increasingly recognised as targets for high impact interventions. We found that much traffic arose from the need to access the sluice room, offices, rest rooms, and separate equipment and storage areas.

Our results indicate that door location had an impact on contamination. For example, the handle used to exit the HDU via Gate 1, to access the operating theatres, was far more contaminated than the handle used the other way when adjusted for frequency of movement. This may be an indicator of ward activity, hand hygiene, or handle design. As expected, we observed a consistently high level of hand hygiene in the operating theatres and this may be the reason for the low level of contamination on the handle used to enter the HDU. In contrast, staff entering the theatre from the HDU (“out” handle) will likely have come into direct contact with high acuity patients in a less controlled environment and may have found it more difficult to maintain such high levels of hand hygiene compliance. This however may not be the full story. The average contamination per movement was highest at this gate and also at Gate 10, which connects a MITU side room with the corridor. This may relate to door handle design, as both gates were operated by lever handles.

**Figure 4:**
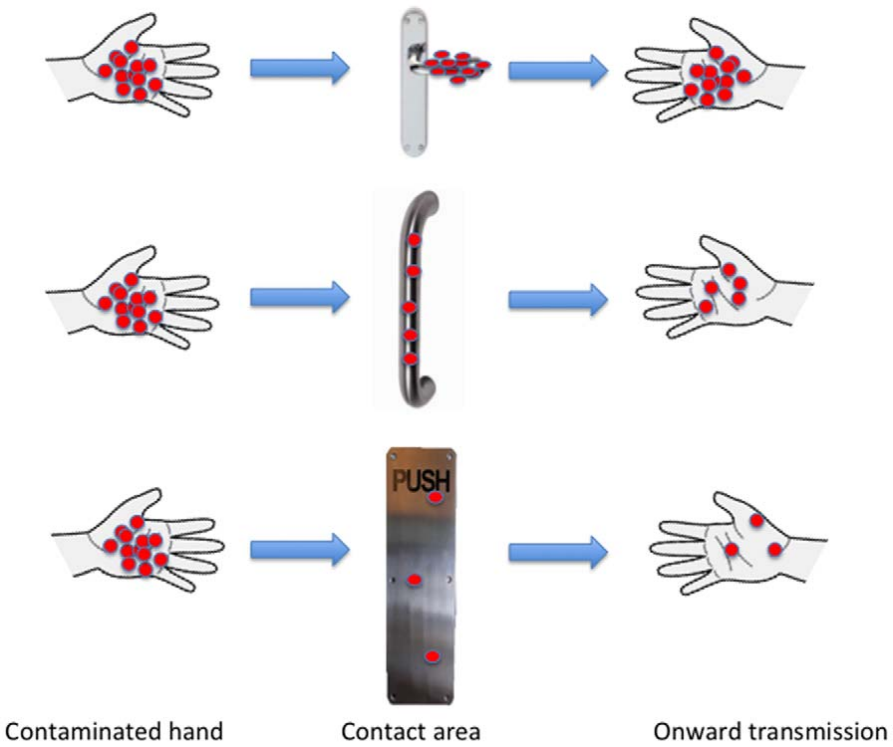
Transmission potential in relation to door handle type.

Door handle design may also have contributed to the TVC/movement results for Gates 4,5 and 6. While the hand hygiene facilities were identical on both sides of these three gates, and the activity within the SITU would clearly be greater than outside the SITU, we always observed greater contamination on the “in” pull handle than the “out” push plate. Accepting the variables relating to activity, as discussed above, it is plausible that pull handles “capture' more organisms than push plates. We suggest that this relates to “skin to metal ratio” as illustrated in Figure 4. It would seem logical that door handles that either “capture” a larger proportion of whatever hand contamination is present, concentrate what is captured onto a smaller surface area or both, is a reasonable explanation for our data. The pull handles require grabbing at some point along the vertical bar of the fixed handle, focusing the contact point on the handle and thus reducing the area and concentrating contamination to a small surface. The potential for concentrating microorganisms was even greater on lever handles, where the length of the handle bar is less than one quarter of that of the vertical fixed handle, thereby acting as a smaller lens focussing the microorganisms left behind on contact. Whilst a logical explanation for our findings, we cannot dismiss the possibility that door handle design had no influence on contamination and that sole determinants of contamination were ward activity and hand hygiene.

The design of the healthcare environment is increasing recognised for its impact on health care quality and outcomes [8]–[9]. To our knowledge there is no coordinated study of how people's behaviour is influenced by the built environment and how this relates to microbial spread [10]. We show here that a multidisciplinary approach both reveals the true complexity of microbial spread and the challenge this sets for effective strategies for its control. In the absence of a more ‘intelligently designed’ built environment, recent focus on the near patient space [7] and alcohol based gels has been of great benefit. The WHO recommends undertaking hand hygiene when entering the patient environment. However as staff compliance with hand hygiene is routinely less than 100% [11], introduction of microbes into bed spaces is still a risk. Accordingly, optimising ward design to limit the risk of contamination, is still of value.

Optimising ward design to limit microbial spread is not straightforward and will be determined by many factors such as the existing building if not a new build, limitations on space, and use. In the setting described in this manuscript, we observed that closer, more accessible storage and supply rooms would have resulted in less time spent fetching, carrying and performing mandated handwashing. Closer storage would likely have limited the opportunities for cross contamination and releasing time for direct patient care. In some settings, closer storage of some ward related items may facilitate contamination with patients' flora and this could be undesirable. Whatever the physical and financial constraints and activity demands, we would advocate an informed approach to ward design/modification, to at least consider the implications for the potential for microbial spread. Of particular importance is the area within and around the sluice. We noted high contamination levels on Gate 6, which controlled access to the sluice room. This study did not set out to identify the bacterial species recovered from the door handles. We cannot therefore state whether these organisms were skin commensals, such as coagulase negative staphylococci, transiently carried *S aureus/*MRSA, or faecal organisms such as *E coli*. If the latter were predominant, it would indicate that the high levels of contamination emanated from the sluice. The sluice room represents a potentially problematic area where a door is desirable to help limit the spread of faecal organisms while also providing surfaces, such as the handles, which could facilitate organism transmission.

There are very limited data on door handles and their potential for microbial transmission. In a study looking at surrogate markers of nosocomial pathogen transmission, door handles were highlighted as one site that rapidly became contaminated within the context of a neonatal intensive care setting [12]. A recent study has shown that it is possible to reduce bacteria on door handles provided they are regularly cleaned. Even with regular cleaning, bacteria were detected on more than 20% of handles [13].

Cleaning, both of hands and the environment, has been widely accepted as an important factor in curbing the spread of pathogens in hospitals [14]. Our data indicate that, while cleaning is important, it is not always practical, as in some cases a single touch by a contaminated hand was sufficient to result in a confluent plate. A potentially innovative approach to limiting environmental contamination is the use of spontaneously antimicrobial surfaces. Of these, copper-based microfibre cleaning systems [15] or copper furnishings look particularly promising, although the latter are expensive and still in need of regular cleaning [16].

The layout of the units, variably and constantly contaminated by the sick patients they contain, can therefore support or undermine policies designed to limit the spread of infection as well as enabling healthcare staff to work more effectively. The use of automatic doors or the elimination of doors altogether could be a solution to reducing the dissemination of microorganisms acquired from door handles, although should be weighed up against the potential for airborne transmission and the importance of visually defined thresholds, themselves prompting hand hygiene. Our findings offer a possible explanation for Cepeda *et al's* surprising findings that side room use in the context of ICUs failed to reduce the rate of MRSA cross-infection [17]. This, however, is only one of a number of healthcare design features that could be considered to optimise effective delivery of care and control of healthcare associated infections.

Architects may not have the necessary information or knowledge available to inform optimal healthcare design as regards the spread of infection. Whilst door handle design may appear trivial at the design stage and largely ignored, it is one of many “trivial” design features that might silently undermine microbial transmission control. Novel door handles are being developed and may prove to be more ‘resistant’ to microbial contamination than existing designs. The multidisciplinary approach taken in this study could serve as a paradigm for future healthcare design. A network of architects, engineers, microbiologists, nurses doctors and hospital administrators working together at multiple stages of the design process could achieve those efficiencies seen in car and kitchen design and manufacturing. These synergies between providers of healthcare and those responsible for the buildings in which it is delivered would seem essential for better, evidence based and optimal healthcare building design.
